# Tough and Self-Healable Nanocomposite Hydrogels for Repeatable Water Treatment

**DOI:** 10.3390/polym10080880

**Published:** 2018-08-07

**Authors:** Kunhao Yu, Di Wang, Qiming Wang

**Affiliations:** Sonny Astani Department of Civil and Environmental Engineering, University of Southern California, Los Angeles, CA 90089, USA; kunhaoyu@usc.edu (K.Y.); wang259@usc.edu (D.W.)

**Keywords:** tough hydrogel, titanium dioxide, self-healing, heavy metal, dye degradation

## Abstract

Nanomaterials with ultrahigh specific surface areas are promising adsorbents for water-pollutants such as dyes and heavy metal ions. However, an ongoing challenge is that the dispersed nanomaterials can easily flow into the water stream and induce secondary pollution. To address this challenge, we employed nanomaterials to bridge hydrogel networks to form a nanocomposite hydrogel as an alternative water-pollutant adsorbent. While most of the existing hydrogels that are used to treat wastewater are weak and non-healable, we present a tough TiO_2_ nanocomposite hydrogel that can be activated by ultraviolet (UV) light to demonstrate highly efficient self-healing, heavy metal adsorption, and repeatable dye degradation. The high toughness of the nanocomposite hydrogel is induced by the sequential detachment of polymer chains from the nanoparticle crosslinkers to dissipate the stored strain energy within the polymer network. The self-healing behavior is enabled by the UV-assisted rebinding of the reversible bonds between the polymer chains and nanoparticle surfaces. Also, the UV-induced free radicals on the TiO_2_ nanoparticle can facilitate the binding of heavy metal ions and repeated degradation of dye molecules. We expect this self-healable, photo-responsive, tough hydrogel to open various avenues for resilient and reusable wastewater treatment materials.

## 1. Introduction

Wastewater with high concentrations of heavy metal ions or dye molecules has been a ubiquitous problem for environmental sustainability and human health [1,2,3,4,5]. Dye molecules or heavy metal ions may transit to highly toxic products in drinking water systems, causing allergy, dermatitis, skin irritations, or even provoking cancer and mutation in humans [6,7,8,9]. Also, the dyes in the water reduce the light penetration and preclude the photosynthesis of underwater green grasses, thus degrading the underwater plant system and destroying the ecological metabolism [10,11,12,13]. Therefore, wastewater must be carefully treated before discharging to the environment. Various methods have been used to treat wastewater, such as adsorption, electrochemical treatment, chemical precipitation, ion exchange, extraction, and filtration [5,14]. Among these methods, the adsorption method is considered as one of the best technologies because the adsorption process is generally effective, convenient, energy-efficient, and inexpensive [3,15,16]. Exiting studies showed that nanomaterials with ultrahigh specific surface areas are promising water-pollutant adsorbents [17,18,19,20]. However, a long-lasting challenge is that the dispersed nanomaterials can easily flow into the water stream and induce secondary pollution [21,22,23]. To address this challenge, we propose to employ nanomaterials to bridge hydrogel networks to form a nanocomposite hydrogel as an alternative water-pollutant adsorber [24,25]. The high porosity of the hydrogel promotes the solute diffusion within the hydrogel matrix. The nanomaterials within the hydrogel matrix can interact with water pollutants to adsorb or degrade those pollutants. Compared to adsorption directly using the nanoparticles, the hydrogel can provide a protecting matrix that constrains the nanomaterials from entering the water stream to induce secondary pollution. Because of their low-cost and ease of fabrication, hydrogels are expected to be excellent adsorbent materials for future large-scale industry applications [26]. Despite their great potential, most of the existing hydrogels that were used to embed nanomaterial agents are relatively weak and brittle. These hydrogels are break easily and are not able to self-heal [24,25]. Also, hydrogels with special chemical groups are responsive to external stimuli (such as temperature, light, magnetoelectric field, or pH value) [27,28]. Harnessing external stimuli to enable click responses of the hydrogel-enabled wastewater treatment is desirable, but still limited [29,30,31].

**Figure 1:**
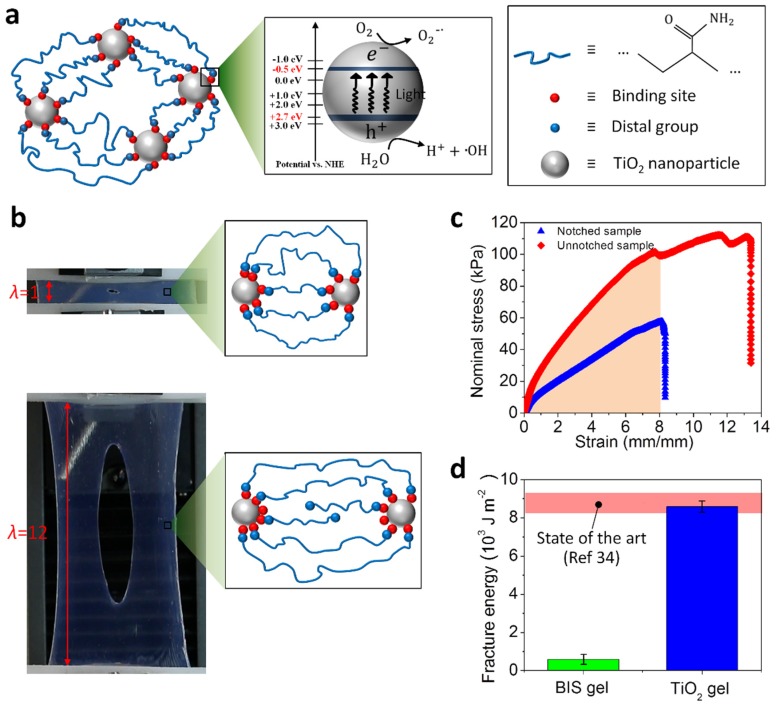
(**a**) A schematic to show the polymer chain network of the TiO_2_ nanocomposite hydrogel and the related light-triggered catalyzing mechanism of the TiO_2_ nanoparticles. (**b**) Stretching of a TiO_2_ nanocomposite hydrogel sample with a small crack. The stretch, λ, represented the length of the deformed sample divided by the length of the undeformed sample. (**c**) Stress-strain behaviors of notched and unnotched samples for a pure-shear test to measure the fracture energy of the TiO_2_ nanocomposite hydrogel. (**d**) The fracture energy of the TiO_2_ nanocomposite hydrogel and a hydrogel with BIS as the crosslinker.

In this paper, we present a tough and self-healable nanocomposite hydrogel that can be activated by ultraviolet (UV) light to efficiently adsorb heavy metal ions and degrade dye molecules in wastewater. This nanocomposite hydrogel is composed of polymer-network-bridged TiO_2_ nanoparticles [32,33]. These TiO_2_ nanoparticles have three functions (Figure 1a): (1) as crosslinkers to bridge polymer chains into three-dimensional networks, which in turn constrain the relative positions of these nanoparticles within the matrix [32,33], (2) as binding agents to adsorb water pollutants such as heavy metal ions and dye molecules, and (3) as photocatalysts to generate free radicals under the UV exposure. Unlike the usual organic crosslinkers that only attach several polymer chains, the TiO_2_ nanoparticle crosslinkers can attach a large number of polymer chains with inhomogeneous chain lengths. When the material is under stretch, the polymer chains are sequentially detached from the nanoparticle surfaces, thus sequentially dissipating a large amount of strain energy and enabling high fracture energy of the material. Also, the detached polymer chains can be re-attached to the particle surface with the assistance of external UV exposure, thus enabling the polymer to be self-healable after fractures. Furthermore, we show that the photo-induced production of free radicals from the TiO_2_ nanoparticles can efficiently facilitate heavy metal adsorption and dye molecule degradation. We expect this self-healable photo-responsive hydrogel to open various possible avenues for resilient and reusable wastewater treatment materials.

## 2. Materials and Methods

### 2.1. Materials

TiO_2_ nanoparticles dispersion (Anatase, 15 wt %, 5–15 nm) was purchased from US Research Nanomaterials (Houston, TX, USA). Acrylamide (AAm, 99%), *N*,*N*-Dimethylacrylamide (DMAA, 99%), *N*,*N*-methylenebisacrylamide (BIS, 99%), potassium peroxodisulfate (KPS, 99%), *N*,*N*,*N*′,*N*′-Tetramethylethylenediamine (TEMED, 99%) and Copper(II) perchlorate hexahydrate (Cu(ClO_4_)_2_·6H_2_O) were purchased from Sigma-Aldrich (Atlanta, GA, USA). Reactive blue 4 (dye content 40 wt %) was purchased from Alfa Aesar (Tewksbury, MA, USA). All chemicals were used as received without further purification.

### 2.2. Fabrication of Nanocomposite Hydrogels

The 10 g TiO_2_ solution was first bubbled with nitrogen for 30 min to remove the oxygen dissolved in the solution. Then, the solution was mixed with 0.039 g (0.009 mol) AAm and 2.079 g (0.021 mol) DMAA under magnetic stirring for 30 min at 20 °C. The mixed solution was cooled down to 0 °C in an ice water bath. 0.1 wt %. KPS and 8 μL TEMED were then added with another 30 min stirring. The obtained solution was poured into a glass tube (diameter 11 mm and length 50 mm) or a glass mold (150 mm × 75 mm × 3 mm) with the cover up to avoid contact with the oxygen. To facilitate the in situ free-radical polymerization, the hydrogel was put in a UV chamber (UVP CL-1000 Ultraviolet Crosslinker, Upland, CA, USA)) with light intensity 37 W/m^2^ (five 8-Watt light bulbs with 254 nm wave length) for 30 min.

### 2.3. Mechanical Tests of Nanocomposite Hydrogels

The fracture toughness of the nanocomposite hydrogel was measured using a pure shear test following [34]. Two identical samples were chosen to carry out the experiments: one hydrogel sample was clamped by rigid plates on an Instron machine (INSTRON, Model 5942, Norwood, MA, USA) with testing domain dimensions of 10 mm × 75 mm × 3 mm; the other sample had the same testing dimensions but with a 30 mm notch in the middle of the sample. Both samples were stretched using a strain rate of 0.06 s^−1^ until rupture. The entire testing time was within 5 min which was much less than the gel de-swelling and healing equilibrium timescale. For the characterization of the self-healing behavior, cylindrical hydrogel samples (diameter 11 mm, length 10 mm) were cut into two pieces with a blade and then were brought into contact with the additional force for 30 s on two sides to ensure the cut surfaces had good contact during the healing process. The samples were then put into a UV chamber with different light intensities (7.4 W/m^2^, 22.2 W/m^2^ and 37 W/m^2^) and controlled moisture using wet paper to avoid the swelling and de-swelling behavior of the samples. The self-healed samples were then stretched uniaxially until rupture using the same testing system (Instron, Model 5942) with strain rate 0.06 s^−1^ at 20 °C.

### 2.4. Light-Triggered Heavy Metal Adsorption

A cylindrical hydrogel (diameter: 11 mm, length: 10 mm) was immersed in a 150 mL beaker containing a 75 mL Cu^2+^ solution (10^−3^ mol/L). NaOH solution (1 mol/L) and HClO_4_ solution (1 mol/L) are used to adjust the pH value of the solution to be around 7 [35]. The beaker was put in the UV chamber with various light intensities (7.4 W/m^2^, 22.2 W/m^2^ and 37 W/m^2^). The concentration of the treated solution was then determined through the solution color assisted by an image processing software Image J (version 1.51). Control experiments were carried out for the same heavy metal solution under the same UV light exposure but without the nanocomposite hydrogel.

### 2.5. Light-Triggered Degradation of Dye Molecules

A cylindrical hydrogel (diameter 11 mm, length 10 mm) was immersed in a 15 mL vial with stopper containing 10 mL blue active dye solutions (0.02 wt %). The bottle was then put in the UV chamber with various light intensities (14.8 W/m^2^, 22.2 W/m^2^ and 37 W/m^2^). The hydrogel was removed from the glass bottle to measure the swelling behavior. The dye concentration of the remaining solution was determined by the solution color using Image J. Control experiments were carried out for the same dye solution under the same UV light exposure but without the nanocomposite hydrogel.

## 3. Results

### 3.1. High Toughness of the TiO_2_ Nanocomposite Hydrogel

The nanocomposite hydrogels were prepared using TiO_2_ nanoparticles as inorganic crosslinkers. After the in situ free-radical polymerization, the gel was formed as a water-mediated three-dimensional network with a schematic shown in Figure 1a. The TiO_2_ nanoparticles and the polymer chains are bonded through reversible bonds, such as hydrogen bonds (between -OH on the particle surface and -NH_2_ group on polymer chains) [33], or ionic bonds (between K^+^ groups from redox initiator KPS and anionic groups of the polymer chains) [36]. Unlike the organic crosslinkers which usually attach only a few polymer chains on one crosslinker, the inorganic crosslinker nanoparticles allow a large number of polymer chains to be attached to the surface of crosslinkers [33,36]. These attached polymer chains do not have the same chain lengths but follow a wide chain-length distribution [37,38]. Under stretching, the short polymer chains are first detached from the particles to release the stored energy in the chains, while the long polymer chains are still attached on the nanoparticle surface to maintain the elasticity of the chain network (Figure 1b). Therefore, under increasing stretch, the polymer chains will be sequentially detached from the nanoparticles to dissipate a large amount of the strain energy. This energy dissipation capability leads to an ultrahigh fracture toughness in the nanocomposite hydrogel. As shown in Figure 1b, the hydrogel sample is stretched with a 10 mm notch in the middle of the sample. When the sample was stretched to 12 times its initial length, the crack in the sample is still blunted without propagating through the sample. To quantitatively measure the fracture energy of the nanocomposite hydrogel, the pure-shear method was employed to test the stress-strain behavior of a notched sample and unnotched sample (Figure 1c) [34,39]. The stress-strain behavior of the notched sample was used to determine the critical strain of the crack propagation, and the area of the stress-strain curve of the unnotched sample under this critical strain is defined as the fracture energy [39]. The fabricated TiO_2_ nanocomposite hydrogels have average fracture energy 8233 J m^−2^, which is over 15 times higher than that of the hydrogel with the same polymer chains but organic crosslinkers *N*,*N*-methylenebisacrylamide (BIS) (Figure 1d). Besides, the fracture energy of the fabricated TiO_2_ nanocomposite hydrogel is comparable to the highest fracture energy of the state-of-the-art tough hydrogels (the pink region in Figure 1d) [34,40].

### 3.2. Light-Assisted Self-Healing

**Figure 2:**
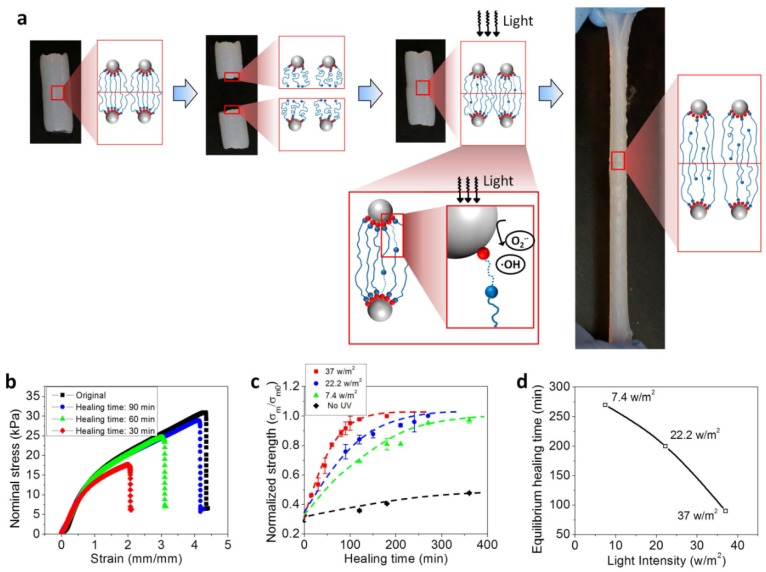
(**a**) Experimental images and polymer-network schematics to show the self-healing experiment. (**b**) Stress-strain behaviors of the original TiO_2_ hydrogel sample and self-healed samples for various healing time. The nominal stress is defined as the tensile force over the original cross-section area. (**c**) The normalized strength of TiO_2_ hydrogel samples as functions of healing time under UV exposure with various light intensities. *σ_m_* and *σ*_*m*0_ denote the strength of the self-healed sample and original samples, respectively. (**d**) Equilibrium healing time of TiO_2_ hydrogel samples as a function of the light intensity. The equilibrium healing time is defined as the healing time corresponding to the normalized healing strength *σ_m_*/*σ*_*m*0_ around 90%.

The TiO_2_ hydrogels exhibit not only high toughness but also extraordinary self-healing capability (Figure 2a). A TiO_2_ hydrogel bar was first cut into two pieces, and then brought back into contact with exposure to UV light for a period of time. Then, the healed sample is stretched until it ruptured. As shown in Figure 2b, the healing strength of the hydrogel increases with an increase in the healing time. When the healing time is long enough, the healing strength reaches a plateau, almost 100% of the strength of the original sample (Figure 2c). However, the strength of the healed sample without the UV exposure is much smaller, less than 50% the strength of the original sample at the plateau (Figure 2c).

The light-triggered self-healing of the TiO_2_ hydrogels can be qualitatively understood as follows (Figure 2a). During the cutting process, the polymer chains around the cutting interface are detached from the particle surface. When two hydrogel parts are brought into contact, the polymer chains with free distal groups diffuse across the interface to find the nanoparticle binding sites to reform the bonding between the polymer chains and the particle binding sites. Effectively, the process can be understood as a coupling of chain diffusion and binding reaction around the interface [38]. Under the UV exposure (wavelength <384 nm, the photons with energy greater than the bandgap of the TiO_2_), electrons are promoted from the valence band to the conduction band leaving holes in the valence band [41,42,43,44]. The photoinduced holes migrate to the particle surface to react with H_2_O to produce hydroxyl radicals (^●^OH) (Figure 1a). At the same time, the conduction band electrons reduce O_2_ to form superoxide radicals ($O_{2}^{-∙}$) (Figure 1a). Like the free-radical polymerization process during the hydrogel fabrication, these free radicals can facilitate the rebinding reaction between the polymer chain distal groups and the particle binding sites (Figure 2a). The acceleration of the rebinding reaction can further promote the chain diffusion across the interface. Therefore, the exposure of UV light as expected, can greatly accelerate the self-healing process (Figure 2c).

To quantitatively verify this mechanism, various light intensities (from 0 to 37 W/m^2^) were carried out for the light-assisted self-healing experiments (Figure 2c,d). According to the mechanism, higher light intensity induces higher concentration of free radicals, thus leading to faster self-healing process. In the experiment, the healing ratio of the hydrogel without UV exposure was found to only reach around 50% at a healing time of 350 min, while the healing ratio of the hydrogel with a small UV exposure (7.4 W/m^2^) reached more than 95% (Figure 2c). Here, the equilibrium healing time is denoted as corresponding to the healing ratio (uniaxial strength of the healed hydrogel over that of the original hydrogel) reaching 90%. Furthermore, the equilibrium healing time was observed to monotonically decrease as the UV light intensity increased from 7.4 to 37 W/m^2^ (Figure 2d).

### 3.3. Light-Assisted Heavy-Metal Adsorption

**Figure 3:**
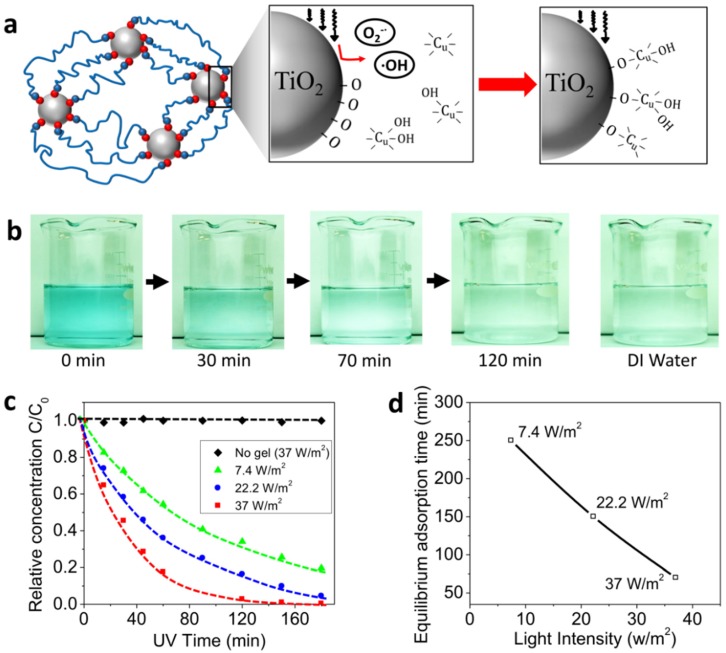
(**a**) Schematics to show the mechanism of the light-assisted adsorption of copper ions (Cu^2+^) on the TiO_2_ nanoparticle surface. (**b**) Image sequences of the treated Cu^2+^ solution after the UV light-assisted adsorption for various adsorption times. (**c**) The relative concentrations of Cu^2+^ as functions of UV exposure time for various UV light intensities. *C* and *C*_0_ denote the concentrations of Cu^2+^ in the treated and original solutions, respectively. (**d**) The equilibrium adsorption time as a function of the light intensity. The equilibrium adsorption time is defined as the UV exposure time corresponding to the relative concentration *C*/*C*_0_ around 10%.

We next studied the light-assisted adsorption of the heavy metal ions with the TiO_2_ nanocomposite hydrogels. The heavy metal adsorption on the TiO_2_ surface in a solution with a pH around 7 is conceptually understood as follows [35,45,46]. The hydroxo complexes among the 10^−3^ mol/L Cu(II) aqueous solution depend on the pH of the solution. For example, Cu^2+^, Cu(OH)^+^ and Cu(OH)_2_ are the main complexes at pH around 7. During surface hydrolysis reactions, the hydrous oxide groups of the TiO_2_ nanoparticles form O–Cu bonds that yield a series of surface Cu(II) complexes such as TiO–Cu^+^, TiO–CuOH^+^ and TiO–Cu(OH)_2_ species (Figure 3a). Besides, the negative surface charges generated by the dissociation reactions at pH 7, similar to the binding between the polymer chain and particle binding site, the formation of the O–Cu bonds can be promoted by the UV induced free radicals (i.e., $O_{2}^{-∙}$ and ^●^OH). Therefore, a higher concentration of free radicals would also induce better performance of the heavy metal adsorption of the TiO_2_ nanocomposite hydrogels.

Here, the TiO_2_ nanocomposite hydrogel samples were immersed in a water solution with heavy metal ions and then the solution was exposed to UV light. The heavy metal ions in the water solution diffuse into the hydrogel matrix and bind on the nanoparticle surface, undergoing a diffusion-binding process. The free radicals accelerate the binding of the metal ions and thus further promote the diffusion of the ions from the solution into the hydrogel matrix. After a period of UV exposure, the hydrogel sample was removed to measure the concentration of the heavy metal ions in the water solution. As shown in Figure 3b, the color intensity of the heavy metal solution decreases as the UV time increases. After UV exposure (37 W/m^2^) for 120 min, the solution color is very close to that of pure DI water (Figure 3b). To quantify the adsorption performance, the heavy metal concentration of the treated water was measured as a function of the UV exposure time (Figure 3c). The measured relative concentration decreased as the UV exposure time increased and reached a plateau when the time was long enough. To verify the experimental results, the control experiments were carried out with the same UV exposure intensity (37 W/m^2^) but without the presence of TiO_2_ nanocomposite hydrogels. The concentration of heavy metal ions was found to remain almost constant over around 3 h of testing time (Figure 3c). Furthermore, various UV intensities were used in the experiments and we found that the adsorption process was significantly accelerated by increasing the UV intensity (Figure 3c,d).

To study the effect of the ions on the property change in the nanocomposite hydrogels, we measured the mechanical and self-healing properties of the hydrogel after the heavy metal ion experiments. Specifically, after the hydrogel was immersed in the heavy metal solution for 2 h under UV illumination, the swollen gel was taken out from the beaker with a volumetric swelling ratio of around 167%. Then, the swollen gel was de-swelled to the same weight and volume as the original gel under a slightly elevated temperature of 40 °C. Compared to the original gel, the obtained gel featured a similar small-strain Young’s modulus, and 81% large-strain shear modulus, 43% higher stretchability, and similar tensile strength (Figure S1). Besides, the obtained gel featured around 88% healing ratio after 2 h healing while the original gel featured around 97% (Figure 2b and Figure S1). Qualitatively, the ionic pollutants may induce minor effects on the mechanical and self-healing properties of the TiO_2_ nanocomposite hydrogels. These minor effects may be because the ionic pollutants slightly alter the chemical equilibrium of the bonding dynamics between the polymer chain and the TiO_2_ nanoparticles.

### 3.4. Light-Assisted Dye Degradation

**Figure 4:**
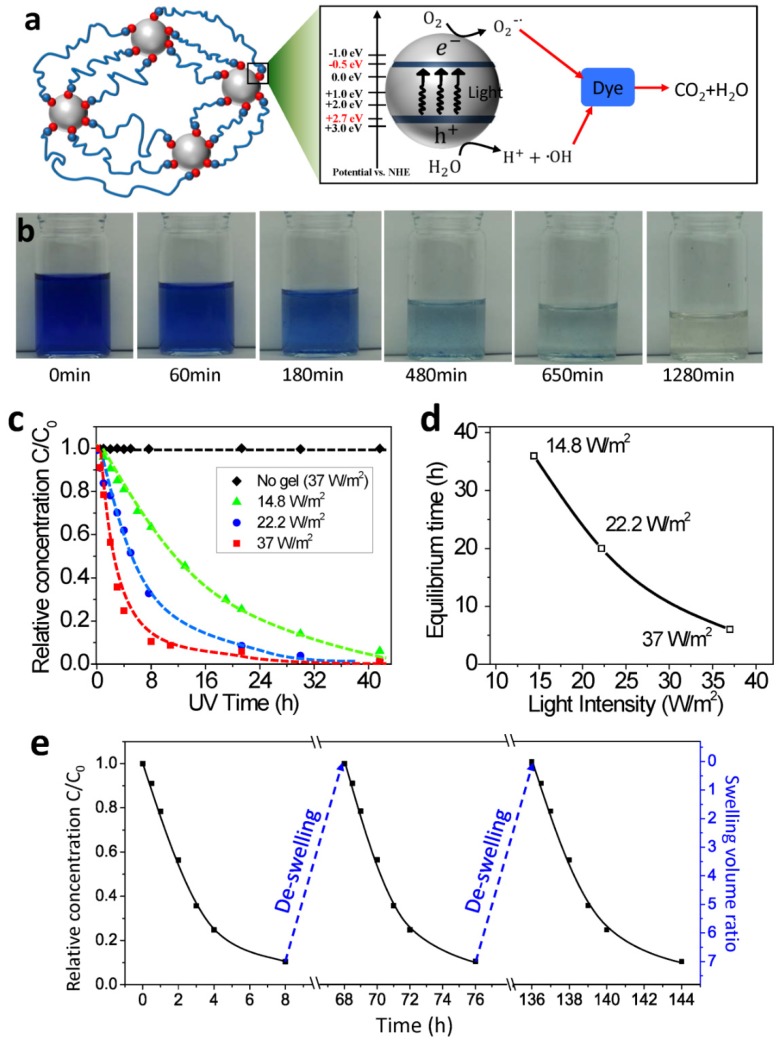
(**a**) Schematics to show the mechanism of light-assisted dye degradation. (**b**) Image sequences of the treated dye solution after the UV light-assisted degradation for various UV exposure time. (**c**) The relative concentrations of the dye molecule as functions of UV exposure time for various UV light intensities. *C* and *C*_0_ denote the concentrations of the dye molecule in the treated and original solutions, respectively. (**d**) The equilibrium degradation time as a function of the light intensity. The equilibrium time is defined as the UV exposure time corresponding to the relative concentration *C*/*C*_0_ around 10%. (**e**) The relative dye concentration and the swelling volume ratio of the hydrogel sample as functions of the processing time.

The TiO_2_ nanocomposite hydrogels not only adsorb heavy metal ions but also adsorb and degrade dye molecules (Figure 4). The mechanism can be understood as follows. With redox initiators potassium peroxodisulfate (KPS) doped on the TiO_2_ nanoparticles, the TiO_2_ nanoparticles are (weakly) positively charged with cations (K^+^). The dye molecule blue active carries a net negative charge due to the sulphonate (SO_3_^−^) groups at the end [36]. When the TiO_2_ nanocomposite hydrogel is immersed in the dye solution, the negatively charged dye molecules migrate into the hydrogel matrix and form weak ionic binding with the positively charged TiO_2_ nanoparticles. At the same time, free radicals (i.e., $O_{2}^{-∙}$ and ^●^OH) are produced on the TiO_2_ nanoparticle surfaces under the UV exposure. These radicals have strong reactions with the dye molecules to decompose the dye into small colorless molecules (H_2_O, CO_2_, and others) (Figure 4a) [47,48,49,50]. The produced radicals decompose the adsorbed dye molecules bound on the nanoparticle surface, and also diffuse through the gel matrix to decompose the freely moving dye molecules.

To test this mechanism, the dye degradation experiments were carried out by immersing the hydrogel samples into a dye solution (0.02 wt% dye) under UV exposure. The initially dark blue color gradually became lighter and lighter, and finally becomes colorless like the DI water (Figure 4b). Quantitatively, the measured dye concentration decreased with increasing the UV exposure time, and eventually reached a plateau close to 0 (Figure 4c). However, the dye concentration in the control experiment with the same UV exposure intensity but without the presence of hydrogel sample remained almost constant over 40 h (Figure 4c). As the UV light intensity increased, the degradation process became more rapid (Figure 4d).

Another outstanding property of using nanocomposite hydrogel to enable dye degradation is that the hydrogel can be used for multiple cycles without lowering the degradation efficiency. Because the binding and degradation agents TiO_2_ nanoparticles are fixed within a hydrogel matrix, the amount of TiO_2_ nanoparticles does not decrease during the degradation process. Besides, after degradation for sufficient time, the amount of dye within the hydrogel matrix decreases to nearly zero; therefore, the efficiency of the second-time degradation is not compromised by the dye adsorption. This is different from the adsorption of heavy metal ions demonstrated in Figure 3: the adsorbed heavy metal ions did not disappear, and the additional adsorption capability of the TiO_2_ gel decreased with the adsorption process. As shown in Figure 4e, a hydrogel sample was first immersed in the dye solution with 8 h UV exposure; the corresponding dye concentration decreased until it reached a plateau with increasing UV exposure time. Then, the swollen hydrogel sample was taken out to de-swell it to the original size for 60 h at 25 °C. After that, the hydrogel sample could be used to degrade a dye solution with the same concentration for a second and third time. The corresponding degradation efficiency was almost the same as that of the first cycle (Figure 4e). It should be noted that the UV-induced radicals may degrade bonds between the polymers and nanoparticles during the dye degradation process. However, due to the reversible character of the bonds, these bonds can reform autonomously. Therefore, the mechanical properties of the used hydrogels in the de-swollen state are not compromised (Figure 4e).

## 4. Conclusions

In summary, we present a tough TiO_2_ nanocomposite hydrogel that can be activated by UV light to demonstrate highly efficient self-healing, heavy-metal adsorption, and dye degradation. Our strategy for the treatment of wastewater harnesses the ultrahigh specific surface area of nanoparticles while demonstrating a novel framework to preclude the negative side effects of the commonly employed nanomaterial-assisted water treatment. Also, external controls with UV light would provide further flexibility in this water treatment strategy. Furthermore, the high toughness and crack-healing capability of the hydrogel matrix offer great robustness of the adsorbent materials. We expect this strategy of stimuli-assisted water treatment with resilient hydrogel materials could be extended to various applications beyond wastewater treatment, such as resilient and pollutant-free artificial organs, tissue dressings [51], contact lenses, soft-material glues [52,53], and hydrogel electronics [54].

## Supplementary Materials

The following are available online at http://www.mdpi.com/2073-4360/10/8/880/s1, Figure S1: Behaviors of nanocomposite hydrogel after heavy metal experiment.
