# Supplementary material for: Tough and Self-Healable Nanocomposite Hydrogels for Repeatable Water Treatment
Source: Polymers (Basel). 2018 Aug 7;10(8):880. doi: 10.3390/polym10080880 (PMC6403828; doi:10.3390/polym10080880)
Supplement: Supplementary file 1 [file polymers-10-00880-s001.pdf]

Supplementary Information for

Tough and Self-healable Nanocomposite Hydrogels for Repeatable Water Treatment

Kunhao Yu, Di Wang, Qiming Wang *

Sonny Astani Department of Civil and Environmental Engineering, University of Southern California, Los Angeles, CA 90089.

*Correspondence: qimingw@usc.edu

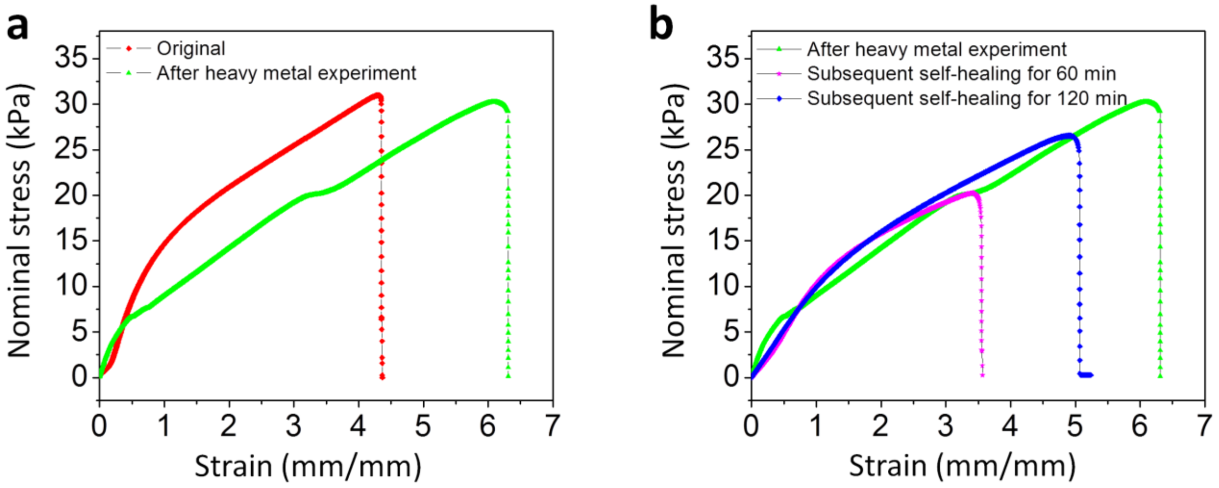

Figure S1. (a) Stress-stress behaviors of the original nanocomposite hydrogel and the nanocomposite hydrogel after the treatment of heavy metal solution for 2 h under UV exposure (light intensity 37 W/m^2). (b) Stress-stress behaviors of the nanocomposite hydrogel after heavy metal experiment and the corresponding healed samples for healing time 60 min and 120 min.
